# Diabetes and restenosis

**DOI:** 10.1186/s12933-022-01460-5

**Published:** 2022-02-14

**Authors:** Scott Wilson, Pasquale Mone, Urna Kansakar, Stanislovas S. Jankauskas, Kwame Donkor, Ayobami Adebayo, Fahimeh Varzideh, Michael Eacobacci, Jessica Gambardella, Angela Lombardi, Gaetano Santulli

**Affiliations:** 1 Department of Medicine, Einstein Institute for Aging Research, Einstein-Mount Sinai Diabetes Research Center (ES-DRC), The Fleischer Institute for Diabetes and Metabolism (FIDAM), Albert Einstein College of Medicine, New York, NY USA; 2 Department of Molecular Pharmacology, Wilf Family Cardiovascular Research Institute, Institute for Neuroimmunology and Inflammation (INI),, Albert Einstein College of Medicine, New York, NY USA; 3 International Translational Research and Medical Education (ITME) Consortium, Department of Advanced Biomedical Sciences, “Federico II” University, Naples, Italy

**Keywords:** ACS, BMS, CABG, DES, Diabetes, Endothelial dysfunction, Epidemiology, Hyperglycemia, PCI, Restenosis, STEMI, Stent, VSMC

## Abstract

Restenosis, defined as the re-narrowing of an arterial lumen after revascularization, represents an increasingly important issue in clinical practice. Indeed, as the number of stent placements has risen to an estimate that exceeds 3 million annually worldwide, revascularization procedures have become much more common. Several investigators have demonstrated that vessels in patients with diabetes mellitus have an increased risk restenosis. Here we present a systematic overview of the effects of diabetes on in-stent restenosis. Current classification and updated epidemiology of restenosis are discussed, alongside the main mechanisms underlying the pathophysiology of this event. Then, we summarize the clinical presentation of restenosis, emphasizing the importance of glycemic control in diabetic patients. Indeed, in diabetic patients who underwent revascularization procedures a proper glycemic control remains imperative.

## Introduction

The global burden of cardiovascular disease is disproportionately borne by patients with diabetes mellitus (DM) [1–4]. Hyperglycemia, insulin-resistance, and the increased presence of advanced glycation end products (AGEs) represent a handful of the conditions that contribute to a 2 to fourfold increased risk of both coronary and peripheral artery disease (CAD & PVD) in DM [5–10]. The deleterious effects of these components on the vascular endothelium have been shown in the literature to be closely associated with macrovascular disease including diffuse atherosclerosis [11–13]. However, it is the complications of diabetes-associated heart disease—including vascular occlusion, restenosis, and in-stent restenosis (ISR)—that make diabetics a particularly complex population to treat.

Restenosis, defined as re-narrowing of an arterial lumen after corrective vascular intervention like percutaneous intervention (PCI) and coronary artery bypass graft surgery (CABG), is an increasingly important issue in clinical practice. Indeed, as the number of stent placements has risen to an estimate of over 3 million annually worldwide, revascularization procedures have become much more common. Unsurprisingly, it has been consistently shown that vessels in patients with DM have an accelerated rate of late loss of lumen diameter and increased ISR [14–16]. In fact, DM is an independent predictor of recurrent restenosis [17–19].

As we have come to realize, the progression of restenosis can be affected by our treatment choices: both for the underlying DM and in the type of intervention in the occluded vessel. With the advent of newer therapies and second-generation drug-eluting stents (DES), restenosis can be better managed than ever before. Herein, we will present a systematic overview of the effects of DM on ISR after coronary angioplasty. We searched in PubMed original clinical studies presenting in the title the words diabetes, in-stent restenosis, restenosis; studies not in English and abstracts were not included.

### Effects of diabetes on the cardiovascular system

Despite the widespread use of hypoglycemic agents and greater awareness, diabetic patients experience significantly higher all-cause and cardiovascular mortality rates than subjects without diabetes after adjustment for other risk factors (16% and 18%, respectively in 2019) [20, 21]. Macrovascular complications mediated by atherosclerosis prove to be the leading cause of premature death in this population [22–24]. Nonetheless, microvascular complications can often present clinically in the form of diabetic nephropathy, neuropathy, and retinopathy [25–32]. A major contributor to both types of vasculopathy is endothelial damage, mediated in part by the actual glycemic control of each patient [33]. Hyperglycemia can contribute to oxidative stress through the production of mitochondrial superoxide, NADPH reduction through polyol accumulation, and AGE synthesis through the nonenzymatic oxidation of glycoproteins—all of which are capable to cause damage to the endothelial cells; the vascular endothelium is particularly sensitive to the effects of hyperglycemia since endothelial cells do not adaptively downregulate their GLUT-mediated uptake of glucose [34–37].

Diabetic cardiomyopathy represents the direct effect of diabetes on both the structure and function of the heart [7, 38, 39]. Even after adjustment for conventional risk factors (like age, CAD, dyslipidemia, and hypertension), those with diabetes have a markedly higher risk for the development of heart failure [7]. This cardiomyopathy typically presents left ventricular hypertrophy and diastolic dysfunction at the echocardiographic examination, often leading to heart failure with preserved ejection fraction (HFpEF, ejection fraction ≥ 50%) [40, 41]. The mechanisms behind these changes are not fully understood but likely involve many of the processes common to those implicated in vascular endothelial damage, in addition to impaired mitochondrial calcium handling and autonomic neuropathy—all of which have been functionally linked to hyperglycemia [42, 43].

### Pathophysiology of restenosis

#### Definition

ISR, based on its traditional definition, is a ≥ 50% luminal re-narrowing of an artery within or directly adjacent to the stented region after PCI, determined through angiography. The clinical definition of ISR includes the same angiographic criteria along with signs of ischemia and/or acute coronary syndrome (ACS); often requiring target lesion revascularization (TLR) [44]. Finally, recurrent ISR is defined as two or more revascularization failures at the same vascular segment.

#### Overview

The progression of restenosis is gradual, already starting in the early hours after intervention. Using PCI to restore blood flow in atherosclerotic vessels can result in the disruption of the target vessel’s integrity. An intact endothelial lining is an important factor in preventing thrombosis, inflammation, and intimal hyperplasia. ISR occurs as a result of this endothelial damage and subsequent neointimal and vascular smooth muscle cell (VSMC) proliferation [45]. As early as 30 min after endothelial injury, proto-oncogenes have already begun to be upregulated in VSMC nuclei in response to growth factor signaling [46]. These processes form the basis of using pharmacologic agents to reduce cellular growth and migration in a stented vessel. If a stent is not used, however, like in the case of simple balloon angioplasty (BAP), restenosis is primarily mediated by the vessel elastic recoil followed by adverse remodeling [47–49].

Neointimal hyperplasia in patients with diabetes looks phenotypically different to the one observed in non-diabetic patients. VSMC specimens from patients with type 2 DM (T2DM) are phenotypically abnormal and behave in a more aggressive manner (greater adhesion and migration) in cell culture [50, 51]. This process may be partly dependent on the adipokine resistin, which is upregulated in human aortic VSMCs in patients DM [52]. Furthermore, several studies have shown that proinflammatory cytokines (like IL-1β, which is chronically activated in T2DM [53]) are able to induce the change in VSMCs into a secretory state, whereas both glucose and insulin could increase VSMC mitogenesis [54, 55]. These findings are consistent with accelerated rates of coronary narrowing and thrombosis in T2DM and highlight the importance of a tight glycemic control in these patients.

De novo neo-atherosclerosis may also be present in the site of the lesion, the progression of which—mainly mediated by chronic inflammation and low-density lipoprotein (LDL) cholesterol uptake by macrophages [56]—might explain the presentation of unstable angina and thrombotic events in patients years after PCI [57]. Moreover, remnant-like particle cholesterol was shown to be an independent risk factor for ISR [58], whereas high-density lipoprotein (HDL) cholesterol levels are known to be inversely associated with ISR in diabetic patients [59]. Other predictors of ISR include levels of soluble receptor for advanced glycation end products (sRAGE), uric acid, and platelet distribution width [60–63].

Neo-atherosclerosis is suggested to play a critical role in restenosis after the placement of a DES especially if compared to bare-metal stents (BMS) [64, 65]. Indeed, when comparing the two types of intervention, patients given a first-generation DES experienced a significantly earlier and more frequent onset of in-stent neo-atherosclerosis than those with BMS placement [66].

Patients with DM are subject to a unique, rapidly progressive, and widespread form of atherosclerosis, resulting in a higher rate of restenosis after simple BAP [67, 68]. These events increase the chance that a patient with DM will undergo repeated revascularizations, a procedure that has been shown to increase the risk of cardiovascular death fourfold (HR = 4.22; 95% CI, 2.10–8.48) in the 2020 Evaluation of XIENCE versus Coronary Artery Bypass Graft Surgery for Effectiveness of Left Main Revascularization (EXCEL) trial [69].

Impaired endothelial function and increased plasminogen activator inhibitor 1 (PAI**-**1) activity in patients with DM result in a greater risk of late in-stent thrombotic events after PCI, which is already more common with first-generation DES compared to BMS intervention [70]. Moreover, insulin-resistance contributes to increased P_2_Y-receptor-pathway signaling, leading to greater platelet aggregation in DM [71]. These factors underscore the importance of adherence to dual antiplatelet therapy (DAPT, comprising of P_2_Y-inhibitors and aspirin) in patients with DM undergoing PCI. In fact, patients who comply poorly with or cannot tolerate DAPT are likely to benefit more from alternatives to PCI including CABG, BAP, or medical therapy [72].

A major concern in modern stents is delayed failure in the form of late in-stent thrombosis, which is a thrombotic event occurring between 1 month and 1 year after PCI [73]. Although the incidence of late in-stent thrombosis is low (~ 0.35%-0.7% in cases with DES), the outcomes are poor, with a fatality rate of 45% reported in 2005 and a fourfold increase in all-cause mortality (HR = 4.9, 95% CI 1.1–21.4) in 2009 [74–76]. Pathological studies of sirolimus and paclitaxel-eluting stents revealed that localized hypersensitivity against the stent polymer is a major thrombotic factor. Moreover, coronary arteries exhibit a longer delay in healing after DES implantation compared to BMS, consistent with reports of higher rates of very late (> 1 year) in-stent thrombosis in DES [77–79]. Chronic inflammation in response to the DES has shown to be a cause of this phenomenon, evidenced by persistent fibrin deposits and incomplete endothelization, particularly in patients with additional risk factors like DM. In this case, the suppression of neointimal expansion by sirolimus and paclitaxel may be detrimental—impairing the normal healing process of the vascular wall [80]. Thankfully, newer second-generation DES (including zotarolimus and everolimus-eluting stents) are associated with significantly lower rates of early and late in-stent thrombosis compared to sirolimus-eluting counterparts [81]. On top of these thromboresistant properties, everolimus-eluting stents were ranked as the most effective treatment for ISR according to a 2015 meta-analysis of 27 clinical trials in comparison to all other major modalities (in order of effectiveness: drug-coated balloons, first-generation DES, vascular brachytherapy, BMS, BAP, and rotablation) [82].

#### ISR classification and risk factors

There are three main categories of ISR defined by the Mehran System through angiographic classification: Pattern I (focal, ≤ 10 mm length), pattern II (diffuse, > 10 mm length), pattern III (proliferative, > 10 mm, extending beyond the confines of the stent), and pattern IV (totally occluded ISR) [44]. These classifications can be regarded as a measure of a vessel’s intrinsic proliferative response to stent placement. A study published in 1999 found that the long-term need for TLR increases with the higher classes of ISR (HR = 1.7; P = 0.0380) and with the presence of diabetes (HR = 2.8; P = 0.0003) [83]. Taken together with other evidence, DM is suggested to be a strong determinant of neointimal hyperplasia [84, 85]. Other risk factors for ISR include pre-operative variables like vessel diameter, stent length, number of prior stents, age, hypertension, and kidney disease [86–89]. However, post-operative levels of inflammatory markers (including high-sensitivity C-reactive protein, matrix metalloproteinase 2, tumor necrosis factor, and chemokine ligand 2) also serve as potential risk criteria [90–93].

The distribution of neointimal proliferation is most often uniform along the length of the stent but may be focal, as shown by intravascular ultrasound [94]. Various studies have shown that the lesion’s appearance can vary significantly with the type of stent used: notably, restenosis in BMS is generally more diffuse than in DES [95]. On the other hand, in-stent neointimal proliferation in patients with DM tends to be located more towards the edges of the stent [44].

Lastly, immunologic, genetic and epigenetic mechanisms have been proposed to partakes in the pathophysiology of ISR in diabetic patients [96–124].

#### Epidemiology

The incidence of restenosis varies significantly across studies. Rates have dropped markedly with technological advances in angioplasty. The occurrence of restenosis is estimated to be at about 32–55% in the pre-stent era, 17–41% in the BMS era (after their implementation in the 1980s), compared to less than ~ 18% with the use of second-generation DES [47]. In support of these estimates, there has been a greatly decreased incidence of TLR and cardiovascular mortality in patients using DES over BMS. This estimate includes the use of first-generation sirolimus-eluting stent, in which the occurrence of ISR is comparably low following the procedure but more common in diabetic patients [125–127].

Karl Haase and collaborators determined that the small size of the vessel and the presence of DM were independent predictors for the occurrence of ISR [128]. Although the risk factors for ISR in the general population are well-defined, there have been fewer studies dedicated to the differences between diabetic patients that fall victim to restenosis versus those that do not. A recent prospective study of 920 diabetic patients found that serum VLDL-C and uric acid were associated with a relative risk of ISR after coronary DES implantation of 1.85- and 1.19, respectively [129]. In this study, LDL-C and HDL-C levels were not significantly different between the two groups.

#### Clinical presentation

ISR may present as recurrent signs of myocardial ischemia—often beginning with stable angina pectoris as shown through patient history, stress tests, and ECG modifications [130, 131]. Though, this presentation may be a result of incomplete revascularization or the progression of CAD at a separate site [44]. Rather, the definitive diagnosis of ISR is normally made through coronary angiography [132–135].

Symptoms have been found to develop on an average of about 6 months after PCI in patients with ISR of a BMS. Those with DES generally develop symptoms later, but often between 3 and 12 months and have a more stable symptomatology [44, 136]. Nonetheless, restenosis cannot be seen as a benign condition. ACS arising from ISR is well documented and associated with more adverse cardiac outcomes [137, 138]. A 2014 retrospective study found that of 909 patients undergoing TLR from prior PCI with all generations of stents—including second-generation DESs— the majority presented with ACS at 66–71% [139]. Yet, statistics on the presentation of ISR can vary greatly between studies with many finding that patients with ISR are most commonly asymptomatic or present with stable angina in the DES era [140]. Notably, approximately 50% of patients with restenosis determined initially by angiography have no ISR-related symptoms [141].

### Clinical data

#### Stent efficacy

Ever since Eric Van Belle and colleagues reported no significant increase in ISR risk in diabetic patients in 1997 [142], a preponderant evidence has emerged showing that there is indeed an increased rate of ISR, TLR, ST, and major adverse cardiovascular events (MACE) due to DM when using BAP, BMS, and first-generation DES [68, 143]. Moreover, diabetic patients showed a more heterogenous pattern of the neointima after BMS, resulting in longer high-grade obstruction segments [144]. However, in the second-generation DES era, it remains somewhat controversial [145–151] whether DM (particularly T2DM) is still a statistically significant predictor of long-term adverse outcomes—including ISR, MACE, and late in-stent thrombosis after PCI (Table 1). This uncertainty may reflect the fact that the use of second-generation DES suppresses the difference in outcomes between those with and without DM. Though, the variability may be partly caused by differences in clinical variables (such as secondary prevention), study design, and stent components (including alloy and drug delivery vehicle).

**Table 1:** Relative risk of major adverse cardiac events (MACE), target lesion revascularization (TLR), and stent thrombosis (ST) in patients with and without DM undergoing PCI with second-generation drug-eluting stent (DES)

Study	Study design	Number of diabetic patients	Total patients	T1DM and/or T2DM	Outcome	Relative risk (95% confidence interval)	P-value	References
Konishi et al. (2016)	Observational cohort study Mean follow-up: 958 days	575	1667	T1DM	MACE	1.18 (0.74–1.82)*	0.48	[145]
		199	1291	T2DM	MACE	1.07 (0.77–1.49)*	0.67	
		575	1667	T1DM	TLR	1.92 (1.10–3.29)*	**0.02**	
		199	1291	T2DM	TLR	1.52 (0.97–2.35)*	0.06	
D'Ascenzo et al. (2017)	Retrospective multicenter study Mean follow-up: 650 days	485	1270	T1DM	TLR	**2.0 (1.1–3.6)**	**0.04**	[146]
Honda et al. (2015)	Retrospective single center study Mean follow-up: 23.1 months	713	1669	Both	TLR	1.23 (0.89–1.69)	0.21	[147]
Zheng et al. (2019)	Retrospective single center study Mean follow-up: 325 days and 772 days	133	394	Both	Early TLR	**2.58 (1.29–5.15)**	**0.007**	[148]
					Late TLR	1.56 (0.47–5.21)	0.472	
Pi et al. (2018)	Retrospective multicenter study Mean follow-up: 3 years	1786	4812	Both	TLR	**1.70 (1.22–2.36)**	**0.002**	[149]
					ST	1.55 (0.75–3.21)	0.242	
Kuramitsu et al. (2019)	Retrospective multicenter study Mean follow-up: 4 years	695	1541	Both	Early ST	1.20 (0.81–1.77)	0.36	[150]
					Late ST	1.02 (0.52–1.99)	0.95	
					Very late ST	0.93 (0.51–1.71)	0.83	

Early TLR and late LTR were determined angiographically at the first (325 ± 90 days) follow-up and between the first and second (772 ± 133 days) follow-ups, respectively, in the Zheng et al. study. In the Kuramitsu et al. study, Early ST was classified as occurring within 30 days, Late ST was between 31 and 365 days, and Very Late ST referred to events after 1 year. * = Multivariate analysis used. Bold text = statistical significance at p < 0.05

In any case, there is a clear benefit of using newer-generation stent technology over first-generation DES on ISR when looking exclusively at patients with DM. This finding was shown initially in the Everolimus-Eluting Stent Versus Sirolimus-Eluting Stent Implantation for De Novo Coronary Artery Disease in Patients with Diabetes Mellitus (ESSENCE-DIABETES) prospective trial in 2011 and confirmed by lengthier studies that followed [152, 153]. Notably, four years later, the Taxus Element versus Xience Prime in a Diabetic Population (TUXEDO)–India study demonstrated that there was a significantly greater incidence of TLR (3.4% vs. 1.2%, P = 0.002), in-stent thrombosis (2.1% vs. 0.4%, P = 0.002), and spontaneous myocardial infarction (MI, 3.2% vs. 1.2%, P = 0.004) after 1 year in diabetic patients randomized to the paclitaxel-eluting stent intervention [154]. A more recent retrospective study stratified 13,895 patients with prior MI into normoglycemic, pre-diabetic, and diabetic groups to compare outcomes between first and second-generation DES intervention. The authors found a significantly higher incidence of cardiovascular endpoint and in-stent thrombosis in those treated with first-generation in comparison to second-generation DES within all three glycemic groups [155]. This confirmation is important since prior evidence of the relative benefits of newer DES generations in DM patients post-MI was limited.

When comparing first-generation paclitaxel- and sirolimus-eluting stents specifically in patients with DM, data diverge on the relative risk of MACE of each; these two stent types tend to be comparable in safety profile with sirolimus-eluting stent intervention likely resulting in slightly lower ISR rates over paclitaxel-eluting counterparts according to meta-analyses [156, 157].

Drug-coated balloons (DCBs) and bioabsorbable vascular scaffolds (BVSs) are two modern interventions similar in that they deliver an anti-proliferative agent to the vascular endothelium without permanently adding additional scaffolds. DCBs have proven non-inferior to (and occasionally favorable over) first-generation DES, particularly in coronary small vessel disease. For example, a reduction in TLR was found only in diabetic patients in last year’s Long-term Efficacy and Safety of Drug-Coated Balloons versus Drug-Eluting Stents for Small Coronary Artery Disease (BASKET-SMALL 2) trial [158, 159]. Meta-analysis of the limited studies on DCB in de-novo lesions in diabetic patients presented neutral findings over the use of DES [160]. However, the Restenosis Intra-stent of Bare Metal Stents: Paclitaxel-eluting Balloon vs. Everolimus-eluting Stent (RIBS-IV) trial found that PCI with second-generation DES was associated with lower percent diameter stenosis over DCB (mean follow up of 1 year) [161]; still, the overall need for TLR in both groups was low and comparable, suggesting second-generation DES may be marginally preferable in the context of large vessel disease [161].

The Drug-Eluting Balloon for In-Stent Restenosis (DARE) trial was designed to investigate the relative performance of the paclitaxel-eluting balloon compared with the everolimus-eluting stent (XIENCE) in the treatment of any ISR [162]. In patients with ISR and DM, the Paclitaxel-eluting Balloon resulted in similar 6-months in-segment minimal lumen diameter and comparable rates of major adverse events compared to Xience, and in-segment late loss at 6 months was significantly lower in the Paclitaxel-eluting Balloon arm [163].

Despite the comprehensive evidence for using DES, BMS is still widely used for diabetic patients in the United States [164–167]. This fact may be attributed to both high prices and increased duration of DAPT needed for DES, despite beneficial cost–benefit analysis [168]. Both global and local gaps in access to these new therapies should be viewed as drivers in disparate cardiovascular outcomes between socioeconomic and ethnic groups as demonstrated by countless studies [169–173]. This aspect represents a considerable a problem for patients with diabetes, since they are at a significantly higher risk for revascularization with BMS over DES and tend to be stratified into lower socioeconomic groups [174, 175]. Indeed, these factors must be considered in advocating for patients and informing areas of research in the future.

#### Glycemic control

Glycemic control at the time of PCI plays an essential role in preventing TLR [176–178]. Of note, fasting blood glucose and hemoglobin A_1c_ (HbA_1c_) have been thoroughly investigated as clinical biomarkers for ISR risk [179]. Results between studies often vary regarding the degree to which HbA_1c_ and fasting blood glucose correlated with restenosis in DM and/or metabolic syndrome. However, most authors present evidence that supports the importance of proper pre- and post-operative glycemic control. Recently, in a 2020 cohort study, 420 T2DM patients with DES were given follow-up coronary angiographies and routine HbA_1c_ measurements [180]. In this study, insulin resistance (known risk factor in ISR [181–188]) was correlated with higher tertiles of HbA_1c_ variability and, therefore, may have played a confounding role. Most recently, our group has demonstrated that hyperglycemia plays a decisive role in ISR, also in non-diabetic patients [189]. Indeed, we showed that admission hyperglycemia increased the risk of ISR at one year follow-up, both in BMS and 2nd generation DES [189]. Intriguingly, this effect was independent from glycemic control.

Hyperglycemia does also affect restenosis in vessels other than the coronary arteries [190–210]. For instance, a retrospective study of 322 patients undergoing carotid artery stenting reported that patients with elevated perioperative fasting blood glucose had significantly less freedom from restenosis at 5 years compared to those with normal fasting blood glucose [211].

#### Hyperinsulinemia and restenosis

Considering the other components of T2DM phenotype, insulin resistance and hyperinsulinemia are likely key players in the increased incidence of restenosis [212]. It is worth noting that multiple studies have linked measures of insulin resistance to the rate of ISR after coronary DES intervention [181, 184, 213, 214]. A 2015 cohort study revealed that the Homeostatic Model Assessment for Insulin Resistance (HOMA-IR)—a model using fasting plasma insulin and glucose [215–218]—could predict the risk of ISR in both diabetic and non-diabetic patients (HR 1.5, 95% CI 1.2–1.8; p < 0.001) [213, 219]. Several studies reported hyperinsulinemia as an independent risk factor for restenosis, even in patients in absence of DM or treatment with insulin [110, 220–222]. Furthermore, restenosis rates are found to be higher in diabetic populations with greater percentages of patients treated with insulin [223]. One explanation for these results is that high insulin levels are directly associated with both increased PAI**-**1 expression, which increases thrombosis and VSMC proliferation (characteristics of restenosis and late ST) [224].

The importance of strict glycemic control on cardiovascular complications in patients with T2DM is well-understood by the biomedical community. A 2009 meta-analysis of five large retrospective studies reported a 15% reduction in events related to CAD (HR = 0.85, 95% CI 0.77–0.93) in patients following a more intensive glucose-lowering regimen [225].

Various studies have suggested that using insulin-sensitizing (IS) strategies like metformin and thiazolidinediones have a more beneficial effect on restenosis than insulin-providing regimens using insulin therapy and sulfonylureas [50]. Moreover, the favorable effects of thiazolidinediones on the progression of restenosis have been demonstrated to be independent of glycemic control; this finding may be explained through their agonistic effect on PPARγ, including the reduction of proinflammatory cytokines, VSMC migration, and neointimal hyperplasia (as measured by carotid arterial intima-media thickness) [226–228].

Clinical data on the effects of IS therapy are somewhat mixed. Numerous small studies have shown that troglitazone, pioglitazone, and rosiglitazone result in reduced restenosis compared to conventional therapy for DM [229–232]. Yet, in 2010, a major randomized control trial of 2368 patients found that those with stable ischemic heart disease and T2DM had a significant reduction in MI only with IS regimens (but not insulin-preserving regimens) post-CABG, with a decreased but non-significant reduction post-PCI [233]. A large retrospective study found that although metformin and thiazolidinediones—individually—did not significantly reduce mortality over the standard of care, co-prescription of the two IS treatments was able to reduce cardiac endpoint (HR = 0.52, 95% CI 0.34–0.82) within 1 year [234]. Further investigation on the clinical efficacy of IS therapy is merited and treatment strategies using combinations of agents may be a promising direction.

#### Pharmacological prevention

Beyond general glycemic control, DAPT and lipid-lowering therapy form the pillars of treatment after the placement of a stent in diabetic patients [235]. Some studies have indicated that the addition of cilostazol to these agents (sometimes referred to as ‘triple antiplatelet therapy’) may decrease the risk of ISR [236–238]. Cilostazol is a vasodilator that suppresses cAMP degradation, a substance whose anti-mitogenic properties have been found to maintain VSMC quiescence in damaged vessels [239]. Reassuringly, a reduction in both late lumen loss and 9-month TLR was observed in cilostazol-treated diabetic patients receiving DES in a recent prospective study [240]. For diabetic patients that cannot receive DES, administration of colchicine has proven to be very effective in reducing the rate of ISR in BMS [241].

The use of antioxidants in ISR has been evaluated for over a decade, taking into account that restenosis is partly mediated by oxidative stress to the vascular endothelium—particularly in presence of hyperglycemia [242–249﻿]. Substances that reduce free radicals like probucol have shown promise in vitro, but are marred by side-effects, including prolongation of QT-interval [250].

While the results of related clinical trials come in, perhaps the most straightforward path to prevention is adequate management of DM itself.

## Conclusions

Available evidence indicates that a tight glycemic control is crucial in diabetic patients who underwent revascularization procedures. Current treatment paradigms for DM should not be cast aside to better manage alterations in insulinemia, but it should be noted that the choice of glucose-lowering agent may affect the chances of developing ISR.

## Data Availability

Not applicable.
